# Longitudinal Analysis Between Maternal Feeding Practices and Body Mass Index (BMI): A Study in Asian Singaporean Preschoolers

**DOI:** 10.3389/fnut.2019.00032

**Published:** 2019-04-02

**Authors:** Phaik Ling Quah, Jing Chun Ng, Lisa R. Fries, Mei Jun Chan, Izzuddin M. Aris, Yung Seng Lee, Fabian Yap, Keith M. Godfrey, Yap-Seng Chong, Lynette P. Shek, Kok Hian Tan, Ciaran G. Forde, Mary F. F. Chong

**Affiliations:** ^1^Singapore Institute for Clinical Sciences, Agency for Science, Technology, and Research, Singapore, Singapore; ^2^Nestlé Research, Vers-chez-les-Blanc, Lausanne, Switzerland; ^3^Department of Obstetrics and Gynaecology, KK Women's and Children's Hospital, Singapore, Singapore; ^4^Department of Pediatrics, Yong Loo Lin School of Medicine, National University of Singapore, Singapore, Singapore; ^5^Division of Paediatric Endocrinology, Khoo Teck Puat-National University Children's Medical Institute, National University Hospital, National University Health System, Singapore, Singapore; ^6^Departments of Paediatrics, KK Women's and Children's Hospital, Singapore, Singapore; ^7^Duke-National University of Singapore Graduate Medical School, Singapore, Singapore; ^8^Lee Kong Chian School of Medicine, Nanyang Technological University, Singapore, Singapore; ^9^Medical Research Council Lifecourse Epidemiology Unit, National Institute for Health Research Southampton Biomedical Research Centre, University of Southampton and University Hospital, Southampton National Health Service Foundation Trust, Southampton, United Kingdom; ^10^Divisions of Pediatric Allergy, Immunology, and Rheumatology, Khoo Teck Puat-National University Children's Medical Institute, National University Hospital, National University Health System, Singapore, Singapore; ^11^Maternal Fetal Medicine, KK Women's and Children's Hospital, Singapore, Singapore; ^12^Department of Physiology, Yong Loo Lin School of Medicine, National University of Singapore, Singapore, Singapore; ^13^Clinical Nutrition Research Center, Singapore Institute for Clinical Sciences, Agency for Science, Technology and Research (A^*^STAR), Singapore, Singapore; ^14^Saw Swee Hock School of Public Health, National University of Singapore, Singapore, Singapore

**Keywords:** maternal feeding practices, comprehensive feeding practices questionnaire (CFPQ), GUSTO, Child BMI z-score, bidirectional associations, Asian cohort, preschoolers

## Abstract

Bidirectional studies between maternal feeding practices with subsequent child weight are limited, with no studies in Asian populations. In longitudinal analyses, we assessed the directionality of the associations between maternal feeding practices and body mass index (BMI) in preschoolers. Participants were 428 mother child dyads from the GUSTO (Growing Up in Singapore Toward healthy Outcomes) cohort. Feeding practices were assessed using the Comprehensive Feeding Practices Questionnaire (CFPQ) at age 5 y. Child BMI was measured at ages 4 and 6 y. BMI and maternal feeding practices subscales were transformed to SD scores and both directions of their associations examined with multivariable linear regression and pathway modeling. Higher BMI at age 4 was associated with lower encouragement of balance and variety (β = −0.33; 95%CI: −0.53, −0.13), lower pressure to eat (β = −0.49; −0.68, −0.29) and higher restriction (β = 1.10; 0.67, 1.52) at age 5, adjusting for confounders and baseline feeding practices at 3 years. In the reverse direction, only pressure and restriction at age 5 were associated with lower and higher child BMI at age 6 years, respectively. After the adjustment for baseline BMI at age 5, the association with pressure was attenuated to non-significance (β = 0.01 (−0.01, 0.03), while the association with restriction remained significant (β = 0.02; 0.002, 0.03). Overall, associations from child BMI to maternal restriction for weight control and pressure feeding practices was stronger than the association from these maternal feeding practices to child BMI (Wald's statistics = 24.3 and 19.5, respectively; *p* < 0.001). The strength and directionality suggests that the mothers in the Asian population were likely to adopt these feeding practices in response to their child's BMI, rather than the converse.

**Clinical Trial Registry Number and Website**

This study was registered at clinicaltrials.gov as NCT01174875 (www.clinicaltrials.gov, NCT01174875).

## Key Messages

- Children's BMI is associated with feeding practices of restriction and pressure, but only the feeding practice of restriction is associated with subsequent BMI outcomes, not pressure.
- The strength of associations was stronger from child BMI to maternal feeding practices
- Mothers in the Asian population were likely to adopt these feeding practices in response to their child's BMI, rather than the converse.

## Background

Childhood obesity is on the rise in many Southeast Asian countries (1), and feeding strategies such as maternal feeding practices have been proposed to modify a child's food intake, and potentially influence the weight status of the child (2, 3). The most well-studied maternal feeding practices in association with child body mass index (BMI) are controlling feeding practices, such as pressuring a child to eat more (“pressure”), restricting certain foods (“restriction”), and monitoring intakes of high-sugar and high-fat foods (“monitoring”) (2).

Prospective studies have found that higher use of restriction (4), but lower use of pressure to eat (5) were associated with higher BMI, while monitoring feeding practices were not associated with subsequent BMI (4, 6). There are also studies supporting a “child-responsive” model where it is hypothesized that parenting strategies such as feeding practices are being driven by their child's characteristics (weight status, eating behavior): higher child BMI was associated with less parental monitoring and pressure to eat (7, 8), but higher parental control (8–10) and restriction (10, 11). Further support of this particular model came from a recent study proposing that the degree to which parents limit or encourage children's food intake is also partly driven by children's genetic predispositions to higher or lower BMI (12). Consequently, based on current evidence, it is possible to hypothesize that the maternal feeding practices-child BMI relationship could be bidirectional.

An increasing number of longitudinal studies have explored relationships between maternal feeding practices and BMI in Western populations (5, 7–10). To our knowledge, no longitudinal studies have been conducted in South East Asian populations, where feeding practices could be influenced by different factors, or lead to different outcomes because of the cultural contexts (13–15), and more evidence is needed to tailor interventions to the specific needs of these populations (5, 8, 10). Lindsay et al. highlighted the paucity of studies between feeding practices and BMI in South East Asian preschooler-aged children, where only 3 studies were published, 2 in Thailand and 1 in Vietnam and all were cross-sectional (16). There is also evidence suggesting differences in the feeding practices of South Asian mothers compared to other ethnic groups: Asian mothers were reported to use more authoritarian parenting styles (15), and controlling feeding practices like “pressure to eat” compared to Caucasians mothers in the United States (14, 15). Differences in these practices may also affect child weight outcomes differently; Cachelin et al. found that lower levels of parental control were associated with childhood obesity only in the Asian mothers but not in other ethnic groups (Latino, African and Caucasian) in the United States (13). Chinese parents and grandparents in Asia have also been reported to be more likely to underestimate boys' weight status compared to girls (17–19). In Singapore, sex differences were found to influence parental feeding approaches, where among girls, parents tended to have more frequent prompts to eat, restrict food or practice general controlling feeding practices. Singaporean parents have also been observed to be more likely to underestimate the weight of their overweight children suggesting a preference for “chubbier” children (20). These cultural perceptions itself could lead to differences in feeding practices to control energy intake leading to future weight problems.

Furthermore, studies have yet to examine other feeding practices that have previously been shown to be associated with children's dietary intakes or BMI in cross-sectional studies (21–23). For example, using food as an emotional regulator or reward, and allowing a child full autonomy over their feeding environment have been associated with overconsumption of calories, and possibly higher child BMI in western populations (24, 25). Additionally, parental modeling of healthy food intake, creation of a healthy diet and home environment may also be helpful for promoting optimal BMI in children (26). There are currently no longitudinal studies examining if these feeding practices are associated with subsequent child weight, or vice versa.

Maternal feeding practices at the age of 5 years is a key period for parents to teach children about making good decisions about their diet as they progress from being completely dependent on their parents, to developing more autonomy in feeding (27). Furthermore, observational studies have shown that maternal feeding practices are stable at ages 5 and 6 years (28, 29), while a randomized controlled trial has shown the effectiveness of a parenting intervention for children in this age group (30). Lastly, understanding the potential influence of feeding practices on BMI is important at this age since changes to BMI during this period have been suggested to be the strongest predictors of BMI in adolescents (31).

This study is the first to be conducted in a mother-offspring Singaporean cohort with a diverse multi-ethnic population. We first aim to conduct an exploratory analysis between the 12 different maternal feeding practices subscales and child BMI: using maternal feeding practices measured at 5 years and child BMI at both 4 and 6 years of age, we could examine bidirectional associations. Our second aim would be to use only selected feeding practices which were significantly associated with BMI to test the strength of the bidirectional associations.

## Methods

### Study Design and Participants

We obtained data from the Growing Up in Singapore Toward healthy Outcomes (GUSTO) cohort study (www.clinicaltrials.gov, NCT01174875). Detailed information on study design and measurements has been previously published (32). Eligible participants were of Chinese, Malay, or Indian ethnicity with homogenous parental ethnic background, and intended to deliver either at National University Hospital or KK Women's and Children's Hospital. Of 3,751 women screened, 2,034 met eligibility criteria, and 1,247 were recruited (32). Ten twins, 85 IVF cases and 70 dropouts and loss-to-follow-up participants were excluded from the 1,247 women recruited to the study. The IVF cases from this study were excluded due to their potentially different growth trajectories from those who were conceived naturally (33, 34). Women who were on chemotherapy or psychotropic drugs or those with type 1 diabetes were excluded. The National Healthcare Group Domain Specific Review Board and the SingHealth Centralized Institutional Review Board provided ethical approval for the GUSTO study. All participants provided written informed consent. This study was registered at clinicaltrials.gov as NCT01174875.

### Measures

#### Assessment of Maternal Feeding Practices

When the child was age 5 years, we administered the CFPQ (35) to the mothers in English, which is the official working language in Singapore. Mothers who required the questionnaire to be translated to other languages such as Mandarin, Malay or Tamil were excluded from this particular study. This questionnaire comprises 49 items answered using two response formats depending on whether the items addressed frequency or degree of agreement. The response formats are “never, rarely, sometimes, mostly, always” or “disagree, slightly disagree, neutral, slightly agree, agree” (35). In total, 12 subscales were measured: Encourage balance and variety (4 items, e.g., “I encourage my child to try new foods”), Environment (4 items, e.g., “Most of the food I keep in the house is healthy”), Involvement (3 items, e.g., “I involve my child in planning family meals”), Modeling (4 items, e.g., “I model healthy eating for my child by eating healthy foods myself”), Monitoring (4 items, e.g., “How much do you keep track the sweets that your child eats?”), Teaching about nutrition (3 items, e.g., “I discuss with my child the nutritional value of foods”), Child control (5 items, e.g., “Do you let your child eat whatever s/he wants?”), Emotion regulation (3 items, e.g., “When the child gets fussy, is giving him/her something to eat or drink the first thing you do?”), Food as reward (3 items, e.g., “I offer my child his/her favorite foods in exchange for good behavior”), Pressure (4 items, e.g., “My child should always eat all of the food on his/her plate”), Restriction for health (4 items, e.g., “I have to be sure that my child does not eat too much of his/her favorite foods”), and Restriction for weight control (8 items, e.g., “I encourage my child to eat less so he/she won't get fat”) (35). Subscale scores were calculated using only fully completed questionnaire items (no missing data) from each subscale.

This questionnaire has been validated among parents of children aged 18 months to 8 years (35), and is suitable for use in this age group. It has also been validated for use in a Malaysian population (36)—a country in South East Asia which is in very close proximity, and with similar ethnic groups to Singapore. Furthermore, confirmatory factor analysis has shown that the CFPQ used in this study fit our data moderately according to fit statistics provided by Hu and Bentler (37): χ 2, (df = 836) = 2,318, *p* < 0.001, RMSEA = 0.057 (PCLOSE < 0.001), SRMR = 0.070, CFI = 0.837, and TLI = 0.816. The calculated Cronbach's alpha of all the subscales based on the current dataset range from 0.56 to 0.86 suggesting that the items from the subscales have a moderate to high internal consistency (38). The acceptable Cronbach's alpha value is normally above 0.70. However, values near 0.6 are acceptable especially if the factor only has a few items (e.g., 3 item subscale) (39).

Ideally, maternal feeding practices should have been measured using the same questionnaire (CFPQ) at an earlier time point to represent these feeding practices at baseline. However, we had to use feeding practices assessed by the Preschooler Feeding Questionnaire (PFQ) at 3 years of age to represent baseline feeding practices because the CFPQ was not administered at any other earlier time point. This questionnaire was previously validated for children of preschooler age between 2 and 5 years old (40). Similarly, the confirmatory factor analysis has shown that the PFQ used in this study fit our data moderately: χ2, (df = 1,240) = 2,318, *p* < 0.001, RMSEA = 0.063 (PCLOSE < 0.001), SRMR = 0.105, CFI = 0.694, and TLI = 0.649. The subscales have moderate to high consistency based on Cronbach's alpha that ranges from 0.61 to 0.85 calculated based on the current dataset.

#### Child BMI at 4, 5, and 6 Years of Age

Trained staff measured children's weights and heights by using standardized procedures. The weight of children at 4, 5, and 6 years of age was measured to the nearest 10 g using a calibrated digital scale (SECA model 803; SECA Corp.). Standing height was measured using a stadiometer (SECA model 213). For reliability, all measurements were taken in duplicates and averaged. Child BMI was calculated as weight divided by height squared. Raw BMI alone might not the best measure of overweight in children. However, since BMI varies with age and sex in children, the current BMI presented in this study has been calculated using age- and sex-specific references, and presented as BMI z-scores. The BMI z-scores were calculated using the WHO Child Growth Standards 2007 with the WHO Anthro software (Version 3.2.2) (41), and child overweight/obese were categorized using WHO cut-offs of above +2 z-score for children aged 4, and +1 z-score for children aged 6 years. These WHO cut-offs using BMI Z-scores for childhood overweight and obesity has been shown to be associated cardiometabolic risk markers such as hypertension, high insulin, high HOMA-IR and high triacylglycerol (41, 42).

Age and sex-adjusted BMI z-scores were calculated using the WHO Child Growth Standards 2007 in the WHO Anthro software (Version 3.2.2) (43). Child overweight/obese were categorized using WHO cut-offs of above +2 z-score for children aged 4, and +1 z-score for children aged 6 years.

#### Covariates

Several maternal and infant co-variates were included into the statistical models as potential confounding factors. At the recruitment visit, participants provided data on ethnicity, educational level, household income, and maternal age. Self-reported pre-pregnancy weight was not ascertained for >20% of participants; measured weight at <14 weeks gestation was extracted retrospectively from clinic records, and was used to calculate and represent pre-pregnancy BMI. Mothers' height was measured at 26–28 weeks of gestation using a stadiometer (SECA206, Hamburg, Germany). The BMI at <14 weeks gestation showed a high correlation with pre-pregnancy BMI (*r* = 0.965). From the obstetric records, we extracted information on child sex and birth order. We recorded the child age at CFPQ assessment during the administration of the questionnaire. The duration of exclusive breastfeeding in the first year of life was estimated using data on the frequency and duration of child milk feeding practices collected with interviewer-administered questionnaires at ages 3, 6, 9, and 12 months (44).

### Statistical Analyses

All the 12 CFPQ feeding practices subscales measured at age 5 years were bound to be applied with very different variations in amplitude: To facilitate effect-size comparison between the subscales and to handle positively skewed datasets, the continuous scale scores were transformed into SD scores and summed. Higher scores on a subscale indicate greater use of the particular feeding practice, and the effect size of one unit increase in the scores will be the same across all subscales.

Scores of the 8 subscales from the PFQ measured at age 3 years were calculated in a similar manner to the CFPQ. We used Spearman correlation tests to determine the strength of the associations between the feeding practices subscales at 3 and 5 years of age. Since the PFQ and the CFPQ were not designed to measure the same feeding practices, Spearman correlation values were used to objectively select baseline feeding practices at year 3. Subscales measured at age 3 years using the PFQ that had the highest correlation values, and were statistically significant in correlation (*p* <0.05) to the subscales measured at age 5 years using the CFPQ were selected to represent baseline feeding practice. Pushing the child to eat more at 3 years of age (5 items, e.g., “Made child eat all the food on the plate”) had the highest correlation values to subscales encouraging balance and variety (*r* = 0.13), and pressure to eat (*r* = 0.20) at 5 years of age, and was chosen as a baseline feeding practice. Based on an observation from our cohort study, encouraging balance and variety was associated with increased intake of vegetables in this cohort, which shows that it's quite likely these same parents were also the same ones who were pushing their children to eat more (22). Similarly, concern about child overeating or overweight at 3 years of age (7 items, e.g., “Had to stop child from eating too much”) was chosen as a baseline feeding practice for restriction for weight control at age 5 years because of its highest correlation value (*r* = 0.33) (Supplementary Table 1).

To examine the associations of maternal feeding practices at 5 years with child BMI at ages 4 and 6 years using longitudinal analyses, two sets of linear regression were conducted for each of the 12 subscales from the CFPQ. Only when the associations between feeding practices and BMI of the first and second linear regression analysis were statistically significant after the adjustment for confounders, these specific feeding practices will be selected out of the 12 subscales to be included into the path analysis model. The first linear regression analysis examined the association of child BMI at 4 years with maternal feeding practices at 5 years, and the second linear regression examined the association of maternal feeding practices at 5 years with child BMI at 6 years. For both sets of regression analyses, we examined 3 models: (1) a crude unadjusted model, (2) a model adjusted for confounders, (3) a model adjusted for confounders, and further adjusted for baseline maternal feeding practices at year 3 (only in the child BMI to maternal feeding practices regression analysis), or for baseline BMI z-score at 5 years of age (only in the maternal feeding practices to child BMI regression analysis). The covariates included in both sets of regression analyses were age of CFPQ assessment, BMI during early pregnancy (<14 weeks), child birth order (first child or not first child), duration of breastfeeding (never breastfed, breastfed for <6 months, breastfed for 6 months or more), ethnicity (Chinese, Malay, and Indian), and maternal education (none/primary/secondary or post-secondary/tertiary). The possible covariates in this association were first identified using the direct acyclic diagram (DAG), drawn based on literature review (Supplementary Figure 1). All the final covariates contributed to more than a 5% effect estimate change in the adjusted models compared to the unadjusted models. Furthermore, all covariates except for age at CFPQ assessment were previously reported to be associated with both maternal feeding practices and child BMI outcomes from our GUSTO cohort findings (22, 45, 46). Statistical significance in the linear regression models was identified by a *P*-value of <0.002, determined by applying the Bonferroni corrections accounting for the maximum 24 outcome variables examined in this study, and to minimize type I errors due to multiple comparisons.

We conducted path analysis, similar to the method used by Jansen et al. (8) to assess the strength and direction of association only when the associations of the first and second linear regression analysis were statistically significant after the adjustment for confounders based on the Bonferroni corrections (*p* < 0.002). For each maternal feeding practice, we tested 3 path models separately. Each path model included multiple linear regression models which jointly estimated maternal feeding practices-BMI associations in both directions while accounting for continuity in BMI (at 4 and 6 year of age) over time. All the 3 pathways included in the model accounted for each other, and could directly be compared in strength. As the path model analysis only included maternal feeding practices subscales selected based on significant results of the initial two sets of linear regression analyses, the *P*-value for statistical significance of the path model analysis was set as *p* < 0.05. Wald's test was used to compare whether the child BMI-maternal feeding practices path or the one from maternal feeding practices to child BMI was statistically stronger than the other. This method of analysis was chosen for a typical cross-lagged model for examining bidirectional relationships could not be applied in this study because feeding practices measured using the CFPQ were only conducted at 5 years of age.

Multiple imputations (full conditional specification) were used to account for missing baseline maternal feeding practices (age 3 years), baseline BMI (age 5 years) and covariate values. Frequencies of missing values were <5% for maternal covariates (BMI during early pregnancy (*n* = 19), education (*n* = 3) and duration of breastfeeding (*n* = 10), and slightly higher for maternal feeding practices at 3 years (<13%; *n* = 56). All values were assumed to be missing at random based on the Little Missing Completely at Random (MCAR) test (*P* = 0.11). Missing values were imputed 20 times using multiple imputation analysis, and chain equations. Imputations were based on available information on all exposure and outcome variables included in the study. Analyses were performed on imputed data, and reported effect estimates were pooled results of 20 imputed data sets (47). All analyses were performed using STATA version 14.1 (StataCorp LP, USA).

## Results

**Figure 1:**
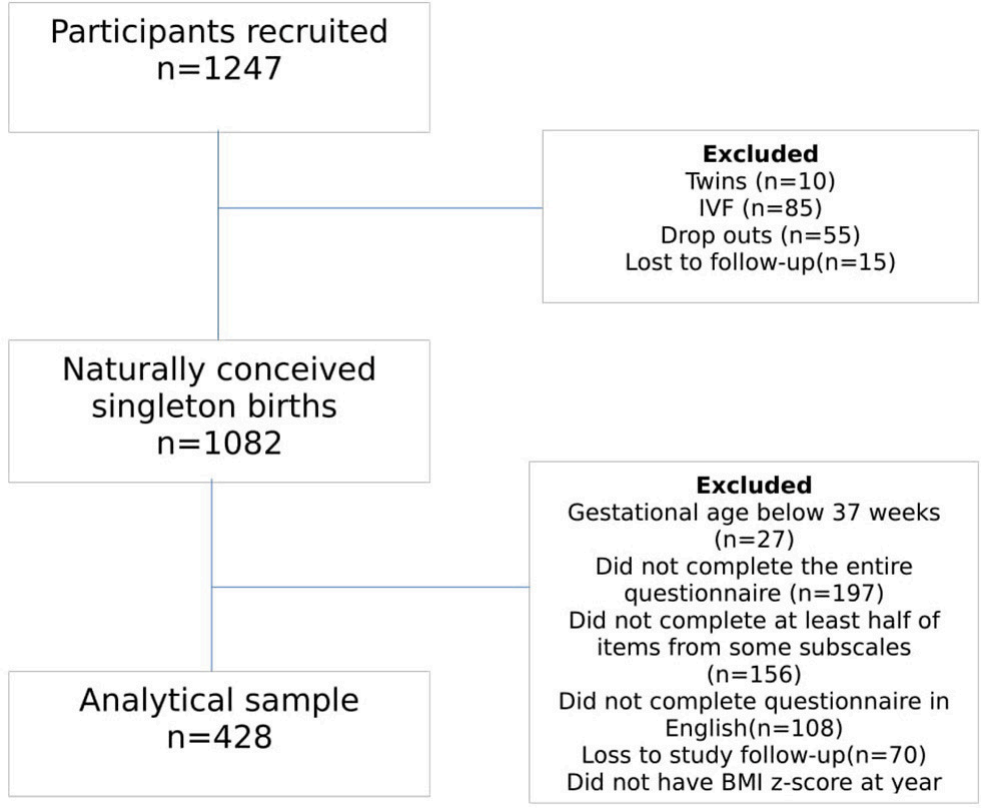
Flow chart of study participants included at timepoints 4, 5, and 6 years old.

From the 1,082 participants with naturally conceived singleton births, we further excluded those lost to study follow-up (*n* = 70), participants who did not complete the entire Comprehensive Feeding Practices Questionnaire (CFPQ) (*n* = 197), those who did not complete at least half of items from some subscales (*n* = 156), those who did not complete it in English (*n* = 108), infants with gestational age below 37 weeks (*n* = 27), and participants who did not have BMI z-score at ages 4 (*n* = 40) and 6 (*n* = 56) years. This yielded a study population of 428 mother and child pairs (Figure 1).

Characteristics of the children and mothers in the study are shown in Table 1. Approximately half the children were boys, seen at a mean age of 60 months, and 44% were first-born. Eight percent of the children were overweight/obese by year 4 and 23% by age 6 years. Forty-seven percent of the participants were of Chinese ethnicity, 32% were Malay and 21% were Indian. Seventy-two percentof mothers were highly educated with post-secondary and tertiary level education; their mean age was 30 years, and 86% had breastfed their infant for <6 months. The mean (SD) mothers' BMI at 15 weeks gestation was 24.1 (5.0) kg/m^2^. Encouraging balance and variety was practiced the most often by mother's with a sum item score of 4.26 (SD = 0.57), and using food as an emotional regulator was practiced the least with a sum of 2.22 (SD = 0.75). A comparison of included (*n* = 428) and excluded (*n* = 724) participants showed no significant differences in demographic characteristics between the groups, except for a lower percentage of Chinese, and higher percentage of Malay and Indian mothers in the group included in the study (*p* < 0.001) (Supplementary Table 2).

**Table 1:** Characteristics of the study population (*n* = 428)^a^.

**Child characteristics**	
Sex (M), *n* (%)	219 (51)
Age at CFQ assessment (years)	4.9 ± 0.2
**Ethnicity**, ***n*** **(%)**	
Chinese	201 (47)
Malay	138 (32)
Indian	89 (21)
**Birth order**, ***n*** **(%)**	
First child	190 (44)
Not first child	238 (56)
BMI SD score at age 4 years	0.22 ± 1.3
BMI SD score at age 6 years	0.09 ± 1.5
Child overweight status at 4 years old (>+ 2 z-score) Overweight, *n* (%)	35 (7.8)
Normal weight, *n* (%)	412 (92.2)
Child overweight status at 6 years old (>+1 z-score) Overweight, *n* (%)	345 (23)
Normal weight, *n* (%)	102 (77)
**MATERNAL CHARACTERISTICS**	
**Educational level**, ***n*** **(%)**	
None/primary/secondary	119 (28)
Post-secondary/tertiary	306 (72)
BMI at 15 weeks (kg/m^2^)	24.1 ± 5.0
Maternal age (years)	30.6 ± 5.3
**Breastfeeding duration**, ***n*** **(%)**	
Never breastfed	15 (3.6)
Breastfed for <6 months	361 (86.4)
Breastfed for >6 months	42 (10.0)
CFPQ subscale scores at year 5 (scores) Modeling	3.70 (0.95)
Encouraging balance/variety	4.26 (0.57)
Healthy environment	3.65 (0.76)
Teaching about nutrition	3.90 (0.70)
Child involvement	3.50 (0.84)
Monitoring	3.52 (1.00)
Restriction for weight control	2.57 (0.85)
Restriction for health	3.71 (0.79)
Pressure	3.60 (0.74)
Emotional regulation	2.22 (0.75)
Child control	2.85 (0.57)
Food as reward	3.05 (0.95)

a *Some data were missing for age at CFPQ assessment (n = 194), BMI at 15 weeks (n = 19), maternal education (n = 3), breastfeeding (n = 10)*.

### Associations Between Child BMI at Age 4 and Maternal Feeding Practices at Age 5

Table 2 shows the results from the first set of linear regression analyses examining the relationships of child BMI at age 4 with maternal feeding practices subscales at age 5. Lower child BMI at age 4 was associated with higher levels of encouraging balance and variety and with pressure-to-eat feeding practices at age 5. The associations remained significant after adjusting for confounding variables, and baseline feeding practice of pushing to eat at age 3 years [encouraging balance and variety: adjusted β = −0.34 (95% CI: −0.54,−0.15); pressure to eat: adjusted β = −0.43 (95% CI: −0.64, −0.22)]. In contrast, higher child BMI at age 4 was associated with higher levels of restriction for weight control at age 5; associations remained significant after adjustment for confounders and baseline feeding practice of concern about child being overweight or overeating at age 3 [β = 1.10 (95%CI: 0.68, 1.52)].

**Table 2:** Linear regression analyses between the child BMI standard deviation (SD) scores at 4 years and maternal feeding practices standard deviation (SD) scores from the CFPQ at 5 years of age^a^.

	**Maternal feeding practices subscales at 5 years of age (SD scores)**					
**Child BMI at 4 years of age (SD scores)**	**Modeling**	***P*-value**	**Encouraging balance and variety**	***P*-value**	**Healthy environment**	***P*-value**
Model 1	−0.24 (−0.48, 0.01)	0.057	−0.33 (−0.53,−0.13)	0.001	−0.02 (−0.22, 0.18)	0.832
Model 2	−0.20 (−0.46, 0.05)	0.120	−0.36 (−0.57, −0.15)	0.001	0.05 (−0.15, 0.25)	0.608
Model 3	-	-	−0.34 (−0.54,−0.15)	0.001	-	-
	**Teaching about nutrition**	***P*****-value**	**Child involvement**	***P*****-value**	**Monitoring**	***P*****-value**
Model 1	−0.14 (−0.30, 0.02)	0.088	−0.12 (−0.28, 0.04)	0.143	0.28 (.016, 0.54)	0.037
Model 2	−0.09 (−0.26, 0.07)	0.253	−0.17 (−0.36,−0.02)	0.025	0.17 (0.10, 0.40)	0.228
Model 3	–	–	–	–	–	–
	**Restriction for weight control**	***P*****-value**	**Restriction for health**	***P*****-value**	**Pressure**	***P*****-value**
Model 1	1.40 (1.00, 1.80)	0.001	0.22 (0.02, 0.42)	0.034	−0.49 (−0.68, −0.29)	0.001
Model 2	1.34 (0.93, 1.75)	0.001	0.24 (0.02, 0.45)	0.031	−0.46 (−0.66, −0.25)	0.001
Model 3	1.10 (0.67, 1.52)	0.001	-	-	−0.43 (−0.64, −0.22)	0.001
	**Emotional regulation**	***P*****-value**	**Child control**	***P*****-value**	**Food as reward**	***P*****-value**
Model 1	−0.07 (−0.25, 0.12)	0.477	−0.20 (−0.43, 0.03)	0.089	−0.08 (−0.25, 0.09)	0.367
Model 2	−0.06 (−0.25, 0.13)	0.520	−0.21 (−0.44, 0.03)	0.086	−0.13 (−0.30, 0.05)	0.164
Model 3	–	–	–	–	–	–

a *All values are β's; 95% CI in parentheses. Values are derived from multivariable general linear regression models. The regression coefficient was interpreted as “For every 1-unit increase in standard deviation scores in the exposure variable, the outcome variable will increase by the beta coefficient value in standard deviation scores*.

Model 1: Unadjusted; Model 2: adjusted for age of CFPQ assessment, early BMI during early pregnancy (15 weeks), child birth order, and duration of breastfeeding, maternal ethnicity, and maternal education; Model 3:Further adjustments were only tested if the confounder adjusted BMI to feeding practices association was statistically significant; further adjustments were conducted only for feeding practice subscale “encouraging balance and variety” and “pressure” with “pushing to eat” at year 3, “restriction for weight control” with “concern about child overeating and overweight at year 3”

*P < 0.004 is statistically significant based on Bonferroni adjustment*.

There were no associations between child BMI and the other 9 feeding practices subscales examined.

### Associations Between Maternal Feeding Practices at Age 5 and Child BMI at Age 6

Table 3 shows the results from the second set of linear regression associations examining the association of maternal feeding practices subscales at age 5 with child BMI at age 6. Higher levels of pressure to eat at age 5 were associated with lower BMI z-score at age 6, after adjustment for confounders [β = −0.09 (95%CI:−0.14,−0.04)]. However, after the adjustment for baseline BMI z-scores at age 5 the association was no longer significant [β = 0.01 (95%CI:−0.01, 0.03)].

**Table 3:** Linear regression analyses between maternal feeding practices standard deviation (SD) scores from the CFPQ at 5y and child BMI standard deviation (SD) scores at 6y (*n* = 428)^a^.

**Feeding practices subscales (SD scores)**	**Child BMI (SD scores)**	***P*-value**	**Feeding practices subscales (SD scores)**	**Child BMI (SD scores)**	***P*-value**
**Modeling**			**Restriction for weight control**		
Model 1	−0.02 (−0.06, 0.02)	0.302	Model 1	0.09 (0.06, 0.11)	0.001
Model 2	−0.02 (−0.06, 0.02)	0.351	Model 2	0.08 (0.06, 0.11)	0.001
Model 3	-	-	Model 3	0.02 (0.002, 0.03)	0.024
**Encouraging balance/variety**			**Restriction for health**		
Model 1	−0.04 (−0.09, 0.01)	0.154	Model 1	0.06 (0.008, 0.11)	0.022
Model 2	−0.05 (−0.10, 0.003)	0.048	Model 2	0.056 (0.005, 0.11)	0.030
Model 3	-	-	Model 3	-	-
**Healthy environment**			**Pressure**		
Model 1	0.012 (−0.04, 0.07)	0.631	Model 1	−0.11 (−0.16,−0.05)	0.001
Model 2	0.02 (−0.03, 0.07)	0.419	Model 2	−0.09 (−0.14,−0.04)	0.001
Model 3	-	-	Model 3	0.01 (−0.01, 0.03)	0.300
**Teaching about nutrition**			**Emotional regulation**		
Model 1	−0.0344 (−0.10, 0.03)	0.319	Model 1	−0.001 (−0.06, 0.06)	0.999
Model 2	−0.02 (−0.08, 0.04)	0.320	Model 2	0.007 (−0.05, 0.06)	0.811
Model 3	-	-	Model 3	-	-
**Child involvement**			**Child control**		
Model 1	−0.05 (−0.11, 0.01)	0.122	Model 1	−0.032 (−0.08, 0.01)	0.145
Model 2	−0.05 (−0.11,−0.001)	0.045	Model 2	−0.02 (−0.07, 0.02)	0.252
Model 3	-	-	Model 3	-	-
**Monitoring**			**Food as reward**		
Model 1	0.06 (0.02, 0.09)	0.001	Model 1	−0.006 (−0.07, 0.06)	0.843
Model 2	0.05 (0.009, 0.08)	0.013	Model 2	−0.01 (−0.07, 0.05)	0.690
Model 3	-	-	Model 3	-	

a *All values are β's; 95%CI in parentheses. Values are derived from multivariable general linear regression models. The regression coefficient is interpreted as “For every 1-unit increase in standard deviation scores in the exposure variable, the outcome variable will increase by the beta coefficient value in standard deviation scores.Model 1: Unadjusted; Model 2: adjusted for age of CFPQ assessment, early BMI during early pregnancy (15 weeks), child birth order, and duration of breastfeeding, maternal ethnicity, and maternal education; Model 3: Further adjustments were only tested if the confounder adjusted maternal feeding practice to BMI association was statistically significant; further adjustments were conducted only for feeding practice subscale “restriction for weight control” and “pressure” with baseline BMI at year 5*.

Higher levels of restriction for weight control at age 5 were associated with higher BMI z-scores at age 6, after adjustment for confounders [β = 0.08 (95%CI: 0.06, 0.11)]. In contrast to the associations observed between pressure to eat and BMI, this association remained significant even after further adjustments for baseline BMI z-scores at age 5 [β = 0.02 (95%CI: 0.002, 0.03)]. There were no associations between the 10 other maternal feeding practices subscales examined and child BMI.

### Strength of Associations Between Feeding Practices and Child BMI

**Figure 2:**
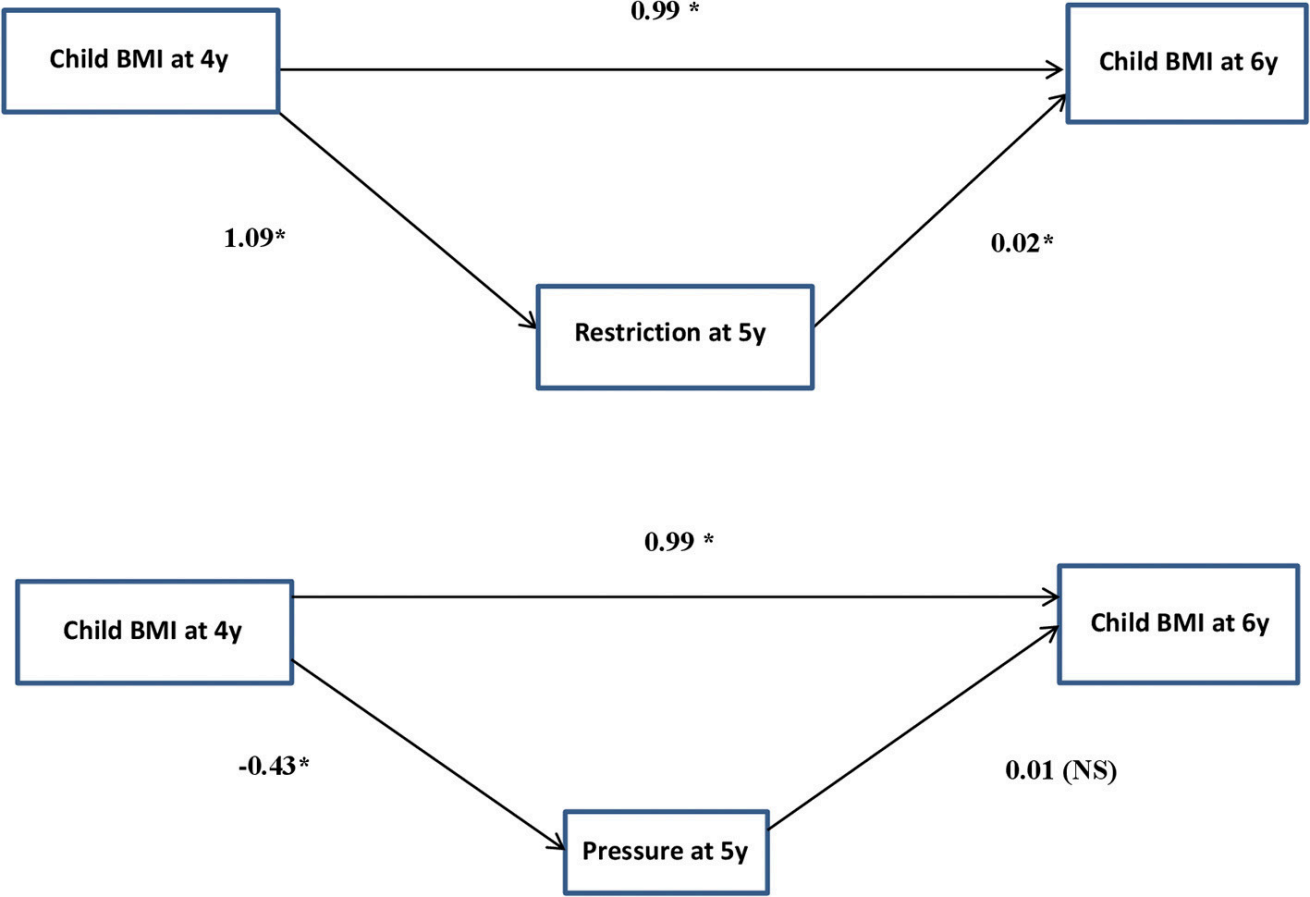
Path models including associations between maternal feeding practices and child BMI in both directions (*n* = 428). Values represent β's derived from linear regression analyses as presented in Table 4 after the adjustment for potential confounders and baseline outcomes. Paths were adjusted for confounders (for age of CFPQ assessment, early BMI during early pregnancy (15 weeks), child birth order, and duration of breastfeeding, maternal ethnicity, and maternal education), and maternal feeding practices at child age 3 y (only in child-BMI at 4y to maternal feeding practices at 5y regressions) and child BMI at age 4 y (only in maternal feeding practices to child-BMI regressions). *p < 0.05 was statistically significant.

Path models were used to determine if each direction of the child BMI and feeding practice association was independent, and to determine the strength of each direction of association independently. Table 4 shows the results of the path analysis, and Figure 2 depicts the pathways in a more intuitive graphic form. Similar to the results from the first and second set of linear regression analyses in Tables 2, 3, higher child BMI z-scores at age 4 was associated with higher restriction for weight control practices at age 5 [β = 1.33 (95%CI: 0.92, 1.75)], and in turn higher restriction for weight control was associated with higher child BMI z-scores at age 6 years [β = 0.08 (95%CI: 0.06, 0.10)], even after adjustment for potential confounders and accounting for the continuity of the BMI z-scores between ages 4 and 6 years (Table 4). The results suggest that the relationship between restriction for weight control and child BMI had a bidirectional association, and Wald's test showed that the direction of association was stronger from child BMI to restriction for weight control (Wald's statistics = 35.1, *p* < 0.001). After the adjustment for baseline outcomes, the effect estimates of both the associations, and Wald's test were attenuated but remained significant [child BMI to restriction for weight control: β = 1.09 (95%CI: 0.66, 1.52); restriction for weight control to child BMI: β = 0.02 (95%CI: 0.005, 0.03); Wald's test = 24.3, *p* < 0.001] (Table 4).

**Table 4:** Path models to determine the strength of associations between maternal feeding practices and child BMI in both directions (*n* = 428): Only for associations that were significant from BMI at 4 years to maternal feeding practices at 5 years, and from maternal feeding practices at 5 years to BMI at 6 years^a^.

	**Stability effect: child BMI from ages 4 to 6 years^b^**		**Child BMI at age 4y to feeding practices at age 5y^b^**		**Feeding practices at age 5y to child BMI at age 6y^b^**		**Comparison of paths: BMI to feeding and feeding to child BMI**	
**Results per feeding practices subscale**	**β (95%CI) for BMI SD score**	***P*-value**	**β (95%CI) for feeding practices SD score**	***P*-value**	**β (95%CI) for BMI SD score**	***P*-value**	**Wald's statistics**	***P*-value**
**RESTRICTION FOR WEIGHT CONTROL**								
Confounder adjusted	0.99 (0.92, 1.05)	0.001	1.33 (0.92, 1.75)	0.001	0.08 (0.06, 0.10)	0.001	35.1	<0.001
Further adjusted for baseline levels of outcome^c^	N.A.		1.09 (0.66, 1.52)	0.001	0.02 (0.005, 0.03)	0.005	24.3	<0.001
**PRESSURE**								
Confounder adjusted	0.99 (0.92, 1.05)	0.001	−0.47 (−0.67,−0.26)	0.001	−0.09 (−0.14,−0.04)	0.001	12.4	<0.001
Further adjusted for baseline levels of outcome^c^	N.A.		−0.43 (−0.63,−0.24)	0.001	0.01 (−0.02, 0.03)	0.529	19.5	<0.001

a *All values are β; 95% CIs in parentheses. Values (except from Wald's statistic) were derived from linear regression analyses. Analyses are also presented in a more intuitive way in Figure 2. Covariates (age of CFPQ assessment, BMI during early pregnancy (<14 weeks), child birth order, duration of breastfeeding, ethnicity, and maternal education) were included in the child BMI–to–feeding regressions and feeding–to–child BMI regressions*.

b Associations were simultaneously included into the pathway model, and the stability effect of child BMI from ages 4 to 6 years was included to account for the continuity in BMI over time

c For the association between Child BMI at age 4y to feeding practices at age 5y, further adjustments were conducted for feeding practice subscale “pressure” with “pushing to eat” at year 3, “restriction for weight control” with “concern about child overeating and overweight at year 3”. For the association between feeding practices at age 5y to child BMI at age 6y, further adjustments were conducted with baseline BMI at year 5.

*P-value < 0.05 was statistically significant*.

For the associations between pressure to eat and child BMI z-score: lower child BMI z-score at age 4 years was associated with higher pressure to eat at age 5 years [β = −0.47 (95%CI:−0.67,−0.26)], and higher pressure to eat at age 5 years was associated with lower BMI at age 6 years [β = −0.09 (95%CI:−0.14,−0.04)]. This suggested that the relationship between pressure to eat and BMI had a bidirectional character, with a stronger direction of association from BMI to pressure to eat (Wald's Test = 12.4; *p* < 0.001). However, after further adjustment for baseline outcomes, only the association from BMI to pressure to eat remained significant [β = −0.43 (95%CI:−0.63,−0.24)], and not that from pressure to eat to BMI [β = 0.01 (95%CI:−0.02, 0.03)], with Wald's test showing an increase in the strength of association from the direction of BMI to pressure to eat (Wald's Test = 19.5; *p* < 0.001) (Table 4).

Sensitivity analysis was conducted was conducted using data before imputation, and the results remain the same (data not shown).

## Discussion

Findings from this longitudinal Asian cohort study in preschoolers aged 4–6 years suggest that the direction of associations from child BMI to restriction and pressure to eat is stronger than the reverse association. This suggests that mothers were more likely to adapt these feeding practices based on their child's weight, as effects on weight changes through these feeding practices were significantly smaller.

Parents exerting restriction on food intake in response to children who were heavier has been reported in a few studies (5, 8, 10, 12). Other studies have had null findings, possibly because of limited statistical power to detect differences with smaller sample sizes (4, 7). We further examined the association in the opposite direction, which showed that restriction was still significantly associated with subsequent child BMI independent of earlier baseline BMI (5 years), suggesting a bi-directional relationship. In contrast with our observations, several other prospective studies have reported null associations between the practices of restriction and child BMI (6–8, 21, 48). However, we have to note that all these studies had at least a 2 year follow-up period unlike our study which had only a short follow-up of 1 year. Interestingly, Farrow and Blissett 2008 have observed that restrictive child feeding practices during infancy (age 1 year) was associated with lower child BMI at age 2 years, and not higher BMI. This highlights how the age group studied might also yield different outcomes. As infants are still primarily fed by their caregivers, a restrictive caregiver would be better able to control the infant's access to high-sugar and high-fat foods compared to children of preschooler age (4). At the preschooler age like our study, children gain more autonomy in feeding, and restrictive feeding might lead to disinhibition and eating in the absence of hunger (28, 49), all of which might lead to higher weight gain.

Our findings on lower child BMI leading to higher pressure to eat feeding practices concur with studies in children with ages ranging between birth (4) to preschool (8, 10), and later childhood (5, 7, 12). The collective evidence highlights how child weight influences the parental practices of pressure to eat, and this seems to be independent of age. When we examined the association from pressure to eat to child BMI, significant associations was attenuated after adjustment for child BMI at age 5. This appears similar to restriction, where the initial BMI of the child seems to influence whether or not the mothers choose to pressure a child to eat. Several other prospective studies have also reported null associations between the practices of pressure and child BMI (6, 7, 21, 48). However, in contrast with our findings are studies that have reported pressure to eat associated with lower weight in children (8, 10). These contradictory findings may be explained by the dissimilar duration of follow-ups in each study. Compared to our study which had a follow-up after just 1 year, the other two studies (8, 10) examined the influence of feeding practices on children's weight only after 2 (8) and 3 years (10). This suggests that a longer follow-up duration might be necessary to observe effects between pressure to eat and weight. Gregory et al. and Rodgers et al. have shown that the use of pressure to eat in children aged 2–4 years old predicted eating behaviors of less interest (48) and enjoyment of food, but more fussiness about food 12 months later (21), but was not related to child BMI.

In addition to restriction and pressure, our study also suggests that a mother's decision to encourage more balance and variety in a child's diet may be influenced by lower BMI in children. The only study supporting our observation is a cross-sectional one conducted in the US and France, where similar findings were observed for both countries (50). However, null associations with subsequent BMI highlights that the use of this feeding practice was largely driven by child weight. From these findings we infer the possibility that mothers who utilize these feeding practices might be more concerned about just altering the diet of the child rather than the weight status of the child (51, 52).

In addition to assessing the possibility of bidirectional associations between feeding practices and child BMI in separate regression models, the longitudinal design of this study enabled us to explore the complex bidirectional associations using a pathway model to determine the strength of the direction of associations. The model supported a relationship between maternal restriction, pressure and child BMI, with the direction from child BMI to restriction and pressure being significantly stronger than the reverse. However, in the case of restriction, parents of children with high BMI were shown to be more likely to use restriction practices, which in turn predict greater weight gain. Our findings might lend valuable information for future weight management interventions in Singapore to help parents replace restriction with alternative feeding practices. It is important to note that restriction for weight, as measured by the CFPQ, primarily assesses restriction behaviors that would be considered “overt” forms of restriction, where parents are directly limiting the child's food intake (e.g., telling the child to eat less). However, it has been demonstrated in the literature that such strategies can actually make these foods even more desirable to the child, leading to overconsumption when the foods become available (53, 54). In contrast, “covert” restriction, in which parents control food access in a way that is not obvious to the child (e.g., not having high-fat and high-sugar foods at home), may lead to more desirable dietary energy intakes, dietary quality, and BMI outcomes (55–57). Lastly, promoting responsive feeding by teaching parents how to recognize and respond to cues of hunger and satiety in children may also be critical for replacing negative feeding practices with more positive ones (58, 59).

Despite conducting the study in a non-Western population, and the differences in cultural setting, our findings were still in line with two other studies in the Netherlands (8) and in Portugal (10) that conducted similar path analyses. Similar conclusions were made that parents may be adapting their feeding practices based the child's weight, rather than the converse. Together, these findings highlight that the practices of restriction and pressure are applied similarly across different contexts and cultures. Furthermore, they indicate the complexity of the relationship between feeding practices and their impact on BMI, where parents might be using a combination of feeding practices instead of focusing on a specific one. Additionally, the contribution of other environmental factors such as physical activity (60) and overall dietary intakes (61) of the child need to be considered. Our findings contribute knowledge to gaps of the current literature by adding to the limited bidirectional studies examining maternal feeding practices and child BMI associations.

### Strengths and Limitations

The strength of the current study lies in the longitudinal design, the larger sample size compared to other longitudinal studies (7, 62) and the repeated measures of body composition which enabled the examination of the maternal feeding practices and child BMI outcomes in both directions.

Furthermore, compared to previous studies, our study explored a broader range of maternal feeding practices, providing a more comprehensive view of other feeding practices that might have bidirectional associations with child BMI. However, our study has some limitations. Firstly, our feeding practices were analyzed by maternal report only, and we have no knowledge of how the reported feeding practices might correspond to actual observed maternal feeding behaviors. Secondly, only 34.3% from the participants recruited in the study were included in the current analysis. There were a higher percentage of Malay and Indian participants included in the analysis of the study compared to the Chinese, which suggested that our findings might not be generalizable to the entire Singaporean population. Thirdly, a longer follow-up duration between feeding practices to child BMI outcomes might be useful to assess if these feeding practices persist over time, and if the effects on weight outcomes are more evident at later ages. It would also have been better if the CFPQ were assessed repeatedly within the same age window to enable an examination of associations in both directions using a cross-lagged model. Lastly, the possibility of residual confounding in this observational study hinders conclusions about causation–temporality does not necessarily mean causality. Maternal BMI has been observed in our previous studies to significantly influence both maternal feeding practices and child BMI (45, 46), and was considered a potential confounder in our analysis. However, future studies should consider focusing on the relationship between maternal BMI and child BMI with maternal feeding practices as a potential modifier in the association.

## Conclusions

This longitudinal study supports a bidirectional relationship between maternal restriction for weight control and child weight status. Overall, the associations from child weight to maternal restriction for weight control and pressure to eat were significantly stronger, suggesting that parents were more likely to adopt feeding practices in reaction to child weight, than the reverse direction where parental feeding practices were driving differences in child weight. These findings inform how we might approach weight management studies in children in the future, especially when focusing an intervention on parental feeding practices and the child's feeding environment in the context of our local Asian population.

## Data Availability

The datasets generated and/or analyzed during the current study are not publicly available due to an ethical restriction (patient confidentiality) which was imposed by the Centralized Institutional Review Board of SingHealth. Interested researchers may request the data by contacting the data team leader of GUSTO at Mukkesh_Kumar@sics.a-star.edu.sg.

## Author Contributions

FY, KG, LS, KT, and Y-SC designed and led the GUSTO cohort study. This sub-study was designed by PQ, MFC, LF, and CF. PQ and JN contributed to the statistical analysis and writing of the manuscript. LF provided intellectual contribution to the write-up of the manuscript and advice on the statistical analysis. MJC cleaned the maternal feeding practice datasets. IA and YL contributed to the anthropometric data collection and the generation of the BMI z-score. PQ, CF, LF, and MFC were responsible for finalizing the manuscript. All authors contributed to and approved the final manuscript.

### Conflict of Interest Statement

KG and Y-SC have received reimbursement for speaking at conferences sponsored by companies selling nutritional products. These authors are part of an academic consortium that has received research funding from commercial affiliations such as Abbott Nutrition, Nestec, and Danone. LF is an employee of Nestec SA, working at the Nestlé Research Center. CF has received reimbursement for speaking at conferences sponsored by companies selling nutritional products, serves on the scientific advisory council for Kerry Taste and Nutrition, and is part of an academic consortium that has received research funding from Abbott Nutrition, Nestec, and Danone.

## Abbreviations

CFPQ: Comprehensive Feeding Practices Questionnaire
BMI z-score: Body Mass Index z-score
PFQ: Preschooler Feeding Questionnaire.
