# Supplementary material for: Longitudinal Analysis Between Maternal Feeding Practices and Body Mass Index (BMI): A Study in Asian Singaporean Preschoolers
Source: Front Nutr. 2019 Apr 2;6:32. doi: 10.3389/fnut.2019.00032 (PMC6454195; doi:10.3389/fnut.2019.00032)
Supplement: Supplementary file 1 [file Table_1.pdf]

Supplementary Table 1: Correlations between subscales encouraging balance and variety, restriction for weight and pressure to eat at 5 years of age with maternal feeding practices subscales at 3 years of age [1].

**Maternal feeding practices at 3 years**	Difficulty in Child Feeding	Concern about Child Overeating or Being Overweight	Pushing the Child to Eat More	Using Food to Calm the Child	Concern about Child Being Underweight
**Maternal feeding practices at 5 years**					
Encouraging balance and variety	-0.08	-0.11*	0.13*	0.01	-0.04
Restriction for weight	-0.15*	0.33*	-0.03	0.04	-0.07
Pressure to eat	0.15*	-0.10	0.20*	0.18*	0.13*

*p<0.05 is statistically significant

[1] The three feeding practices subscales at year 5 were not significantly associated with any of the other subscales of the Preschoolers Feeding Questionnaire (PFQ) at year 3: Child's Control of Feeding Interactions, Structure during Feeding Interactions and Age-Inappropriate Feeding
