# Supplementary material for: Longitudinal Analysis Between Maternal Feeding Practices and Body Mass Index (BMI): A Study in Asian Singaporean Preschoolers
Source: Front Nutr. 2019 Apr 2;6:32. doi: 10.3389/fnut.2019.00032 (PMC6454195; doi:10.3389/fnut.2019.00032)
Supplement: Supplementary file 2 [file Table_2.pdf]

Supplementary Table 2: Characteristics of participants who were included (n=428) and excluded (n=724) from the analysis.

	**Included(n=428)**	**Excluded(n=724)**	**P value**
**Child characteristics**			
Sex (M),n(%)	219(51)	352(53)	0.848
Ethnicity, n(%)			<0.001
Chinese	201(47)	425(59)	
Malay	138(32)	176(24)	
Indian	89(21)	123(17)	
**Maternal characteristics**			
Educational level, n(%)			0.361
None/primary/secondary	119(28)	234(33)	
Post-secondary/tertiary	306(72)	478(67)	
Household income(SGD),n(%)			0.290
0-1999	71(18)	106(16)	
1999-5999	216(54)	393(58)	
>5999	115(29)	175(26)	
Maternal age (years)	30.6 $\pm$ 5.3	30.1 $\pm$ 5.1	0.740
BMI at 15 weeks (kg/m2)	24.1 $\pm$ 5.0	22.5 $\pm$ 3.8	0.138
