# Supplementary figures and images for: Longitudinal Analysis Between Maternal Feeding Practices and Body Mass Index (BMI): A Study in Asian Singaporean Preschoolers
Source: Front Nutr. 2019 Apr 2;6:32. doi: 10.3389/fnut.2019.00032 (PMC6454195; doi:10.3389/fnut.2019.00032)

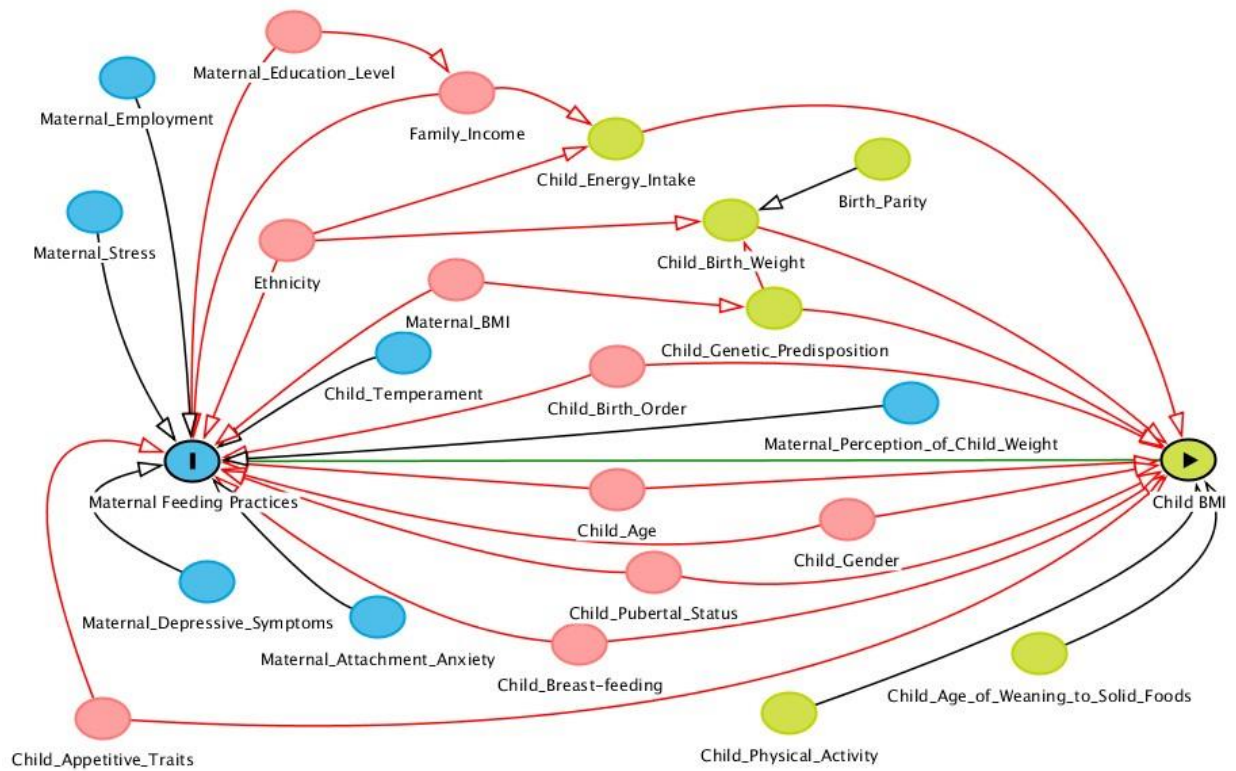

Supplementary Figure 1: The Direct Acyclic Graph (DAG)

Supplement: Supplementary file 3 [file Image_1.pdf]
